# Effects of nickel and cobalt on methane production and methanogen abundance and diversity in paddy soil

**DOI:** 10.7717/peerj.6274

**Published:** 2019-01-17

**Authors:** Tianwei Wang, Zhaoxia Li, Xueping Chen, Xi-En Long

**Affiliations:** 1 College of Resources and Environment, Huazhong Agricultural University, Wuhan, Hubei, China; 2 School of Environmental and Chemical Engineering, Shanghai University, Shanghai, China; 3 School of Geographic Sciences, Nantong University, Nantong, Jiangsu, China

**Keywords:** Nickel, Methane emissions, Archaea, Methanogens, Cobalt, Paddy soil

## Abstract

**Background:**

Paddies are an important anthropogenic source of methane emissions to the atmosphere, and they are impacted by heavy metal pollution. Nickel (Ni) and cobalt (Co) pollution might either enhance or mitigate CH_4_ emission from paddy soils due to the total amounts of metals, bioavailability and functional microbial activity and composition.

**Methods:**

An incubation experiment was conducted, and different Ni and Co concentrations were added to test the effects of trace metals on methane production in paddy soil. The archaea community structure and the abundance of methanogen functional groups in the paddy soil with added Ni and Co were detected using high-throughput sequencing and quantitative PCR based on the 16S rRNA and mcrA (methyl coenzyme M reductase) genes, respectively.

**Results:**

The highest methane production rate was 561 mg CH_4_ kg^−1^ dry soil d^−1^ with the addition of 50 mg kg^−1^ Ni and 684 mg CH_4_ kg^−1^ dry soil d^−1^ with the addition of 25 mg kg^−1^ Co. Accordingly, the *mcr*A gene was most abundant in the 50 mg kg^−1^ Ni addition (3.1 × 10^6^ ± 0.5 × 10^6^ copies g^−1^ dry soil). The lowest *mcr*A gene abundance was detected in the 500 mg kg^−1^ Co addition (9.2× 10^5^ ±  0.4 × 10^5^ copies g^−1^ dry soil). The dominant methanogens were Methanobacterium, Methanosarcina, Methanocella, Methanomassiliicoccus, Bathyarchaeota, and Rice Cluster I (RC-I), and the relative abundances of these groups were higher than 1% in the Ni and Co treatments. Additionally, the archaeal compositions differed significantly in the soils with various Ni and Co additions. The most abundant Methanococcus spp. represented 51.3% of the composition in the 50 mg kg^−1^ Ni addition, which was significantly higher than that of the control (12.9% to 17.5%).

**Discussion:**

Our results indicated that the contamination of soil by Ni and Co significantly affected total methanogens abundance and specific methanogen functional groups. Ni and Co additions to paddy soil promoted methanogenic activity at low concentrations, while they had inhibitory effects at high concentrations. Because paddy soils largely contribute to methane emissions and are increasingly exposed to heavy metal pollution, our results show that future assessments of greenhouse gas flux from paddy soils should take into account the effects of pollution by Ni and Co.

## Introduction

Methane (CH_4_) is a potent greenhouse gas and contributes up to 25% of global warming. Flooded rice fields are the most important sources of atmospheric CH_4_, emitting 10–20% of global methane (Bodelier, 2011). Methanogenesis appears to be the core process of organic matter decomposition in anoxic environments (Goevert & Conrad, 2009; Schauss et al., 2009). This process typically occurs in an oxygen-limited anoxic zone such as paddy soil during the flooding incubation period. In paddy soil, various genotypes of methanogens have been identified, including acetoclastic, methylotrophic, and hydrogenotrophic methanogens (Vaksmaa et al., 2017). Archaea belonging to Rice Cluster I (RC-I) are usually key methanogens in paddy fields (Kögel-Knabner et al., 2010). The diversity of the methanogenic communities has resulted in paddy soil being used as the typical ecosystem to study environmental or anthropogenic effects on methane production and emissions. The CH_4_ production and emission in paddy soil are found to be controlled by several environmetals factors, including fertilizers (Arif, Houwen & Verstraete, 1996) and water regime (Mohanty et al., 2000). However, the availability of trace elements (e.g., heavy metals) required for the growth of methanogens has not been widely researched.

With the rapid development of the mining, metallurgy, foundry and electronic industries, a large amount of heavy metals is released into the soil through irrigation and dry deposition. In China, 19% for the agricultural soils are considered as contaminated based on China’s soil environmental quality limits, mainly with heavy metals and metalloids (The Ministry of Environmental Protection, 2014). Because heavy metals are not biodegradable, they can accumulate in soils for a long time. Some of the metals may be adsorbed to soil organic matter and mineral, and become unavailable to living organisms. Thus, the soluble heavy metals, rather than the total heavy metal content, are considered as the bioavailable part. The bioavailability is also responsible for the toxic effects, determining the proliferation, growth, metabolism and morphology of soil microorganisms (Leita et al., 1995). However, some trace metals, e.g., Ni and Co, play functional roles in the growth and metabolism of microorganisms. The availability of these trace elements could be either too high, causing toxicity, or too low, causing limitations for some microbial processes (Basiliko & Yavitt, 2001). The optimal concentrations of those elements for microorganisms in paddy soils are hard to estimate.

Methanogens and their activity can be affected by heavy metals/trace elements. In pure culture, trace elements (e.g., iron (Fe), nickel (Ni), cobalt (Co), molybdenum (Mo)) are required for optimal growth of various methanogens, including *Methanosarcina barkeri*; *Methanospirillum hungatii*; *Methanocorpusculum parvum*, *Methanobacterium thermoautotrophicum* and *Methanococcus voltae*, and *Methanococcus vanielli*, and *Methanococcoides methylutens* (Demirel & Scherer, 2011). These trace elements play critical roles in biomass synthesis or act as cofactors in metalloenzymes. For examples, Ni or Co ion-containing enzymes involved in methanogenesis have been identified. Methyl coenzyme M reductase, an essential enzyme found in all methanogenic archaea, possesses a nickel-containing cofactor named F_430_. Cobalt is present in cobalamides which play an important role in methylotrophic methanogenesis (DiMarco, Bobik & Wolfe, 1990). However, excessive amount of trace elements would have toxic effects on methanogens and activity.

The requirement of trace metals for the production of methane in biogas digesters has been investigated, highlighting the beneficial and inhibitory/toxic effects of heavy metals (Demirel & Scherer, 2011). The demand of Ni for the methane-forming bacteria and anaerobic digesters has been often reported in literature (Diekert, Weber & Thauer, 1980; Diekert et al., 1981; Speece, Parkin & Gallagher, 1983; Jarrell & Kalmokoff, 1988; Hu et al., 2008). Ni addition stimulates the methane content of biogas, while excessive addition of Ni causes inhibition of methanogenesis. A previous study showed that the optimum/ stimulatory concentrations of Ni varied between 12 mg m^−3^ and 5 g m^−3^ (Takashima & Speece, 1990). Similarly, Co was reported to be a key limiting element for methane production, and the optimum concentrations of Co varied between 5.9 mg m^−3^ and 120 mg m^−3^ for batch cultures of methanogens in a reactor of mono-digestion of maize silage (Lebuhn et al., 2008; Droge, Ewen & Pacan, 2008). In the case of natural paddy soils, only a few studies investigated the effects of trace metal addition on methanogenesis. When two forms of Ni (20 mg kg^−1^) were put in addition to acetate, Ni^2+^ suppressed the induction of the Ni enzyme (hydrogenase, methyl-S-coenzymeM reductase, and carbon monoxide dehydrogenase), while Ni-EDTA enhanced it (Nozoe & Yoshida, 1992). Pramanik & Kim (2014) found only small doses of EDTA application reduced CH_4_ emission by suppressing methanogen activity in paddy soils. These inconsistent results were speculated to be caused by the bioavailability of heavy metals and soil properties. Given that the paddy soil ecosystem is more complex than a bioreactor, it is still unclear how heavy metals actually influence the methanogens activity and community in contaminated paddy soils.

In view of both widespread heavy metals pollution and high level of methane emissions from paddy fields, it is important to determine how metal pollution can influence methane emissions. For example, the main arable land of paddy in China has been polluted with heavy metals, which can affect the methane emissions from these fields (Zhao et al., 2015). However, previous studies on the metal supplementation mainly focused on methanogens biogas digesters. There is no similar study on the effect of high Ni/Co additions on methane production in paddy soil. In addition, soil ecosystems are much more complex than biogas digesters, which might influence the range of metal concentration required for methane production. Thus, the hypothesis of this study was that Ni and Co pollution might either enhance or mitigate CH_4_ emission from paddy soils, which might be due to the total amounts of metals, their bioavailability, and their effects on microbial activity and composition. In this study, laboratory incubations were conducted to analyze the effects of Ni and Co added to the soil. We explored the production of methane (CH_4_) as well as the community composition and abundance of methanogens in the paddy soil.

## Material and Methods

### Rice field sites and soil sampling

Non-contaminated paddy soil was collected from a paddy field located in the Experimental Station of Jiaxing Academy of Agricultural Sciences in Zhejiang Province (30°77′N, 120°76′E). This site is in the center of the Hangjiahu Plain in the Yangtze River Delta. Soil samples were collected from the 20-cm surface layer in three plots without pollution. Five soil cores (diameter: 8 cm, height 20 cm) were collected from each plot, and the 15 cores were mixed together. Part of the soil was stored at 4 °C before the incubation experiments, and another part was naturally air-dried for testing the soil properties. The dried soil was ground through a 2-mm stainless-steel sieve, and the physical and chemical properties of the tested soil were as follows: pH (H_2_O), 6.10; soil organic carbon, 49.7 g kg^−1^; total nitrogen, 2.72 g kg^−1^; ammonia, 1.50 mg kg^−1^; nitrate, 0.34 mg kg^−1^; available Fe, 2.97 g kg^−1^; and sulfate, 0.41 g kg^−1^.

### Incubation experiments

Fresh soil samples (10 g dry soil) were incubated in 120-mL serum bottles, which were sealed with screw-caps with rubber stoppers. Based on China’s soil environmental quality limits, 5 mL of the solution containing different amounts of Ni^2+^ (NiCl_2_, 0, 50, 100, 200 and 500 mg kg^−1^ dry soil) or Co^2+^ (CoCl_2_, 0, 25, 100, 200 and 500 mg kg ^−1^ dry soil) were added. Each treatment has three replicates. The treatments with the Ni additions were referred to as Ni50, Ni100, Ni200, and Ni500; additionally, ck1 referred to the control without Ni nor acetate addition, and ck2 referred to the control without Ni addition but with acetate added. The treatments with Co additions were referred to as Co0, Co25, Co100, Co200, and Co500. Flasks were preincubated at 25 °C for 12 d, and acetate (0.3 mmol g^−1^ dry soil) was subsequently added. The batch experiments of Ni and Co addition were incubated 16 and 21 days after acetate addition, respectively. Methane production potential was determined using gas chromatography (GC-900, Shanghai Kechuang Chromatography Instrument Co. Shanghai, China) with a hydrogen flame ionization detector (FID). Methane concentrations were calculated per kilogram dry weight (g_dw_) of the soil.

The methane production (*P*) was calculated as follows (Wassmann et al., 1998),

(1) \begin{eqnarray*}P= \frac{dc}{dt} \times \frac{{V}_{H}}{{W}_{S}} \times \frac{MW~\times ~{T}_{st}}{MV\times ({T}_{st}+T)} \end{eqnarray*}

where *dc*∕*dt* is the measured change in the mixing ratio of methane in the headspace over time (µmol mol^−1^ d^−1^); *V*_*H*_ the volume of headspace (L), Ws the dry weight of soil (g), MW the molecular weight of methane (g), MV the molecular volume (L), T the temperature (K), and Tst the standard temperature (K). Methane production was expressed as mg CH_4_ kg^−1^ dry soil d^−1^.

### Ni and Co fractionation

To assess the variance in the liability and bioavailability of Ni and Co in the soil after incubation, a sequential extraction procedure was used, based on the methods in Tessier, Campbell & Bisson (1979). In brief, Ni and Co were sequentially extracted using MgCl_2_ (pH 7.0, 1.0 mol L^−1^), NaAc (pH 5.0, 1.0 mol L^−1^), NH_2_OH-HCl (0.04 mol L^−1^), HNO_3_ (0.02 mol L^−1^), HNO_3_-HClO_4_-HF digests, and the extracts were detected using inductively coupled plasma - mass spectrometry (ICP-MS 7500cs, Agilent). These metal fractions were primarily associated with exchangeable, carbonation-bound, iron-bound and manganese oxide-bound, and organic matter-bound to organic matter and phases, respectively.

### DNA extraction and fluorescence quantitative PCR

Soil DNA from each bottle of the different treatments was extracted according to the procedure provided for the Soil DNA Isolation Kit (MoBio UltraClean, San Diego, CA, USA). DNA were extracted in duplicate to avoid the quantification bias due to incomplete and random extraction of soil microbial DNA. DNA quality and quantity were analyzed by a Quawell^®^ Q5000 spectrophotometer (Beckman Coulter, Brea, CA, USA). Fluorescence quantitative PCR was based on the methanogen gene *mcr*A. The functional gene sequences were amplified using the specific primer pair ME1 (5′-GCMATGCARATHGGWATGTC-3′) and ME2 (5′-TCATKGCRTAGTTDGGRTAGT-3′) (Hales et al., 1996). Each reaction (25 µL) was carried out with 12.5 µL of SYBR Premix Ex Taq™ (Takara, Dalian, China), 0.2 µM of each primer and 1-10 ng of DNA. After amplification, a melting curve was generated (65–98 °C, 0.2 °C per read, 6-s hold) to test product specificity. The standard curve was made using a known copy number of plasmids with ten-fold serial dilution, which included the *mrcA* fragments from *Methanosarcina* that were ligated to the vector (p-GEM T-easy, Promega, Madison, WI, USA).

### High-throughput sequencing

The archaeal 16S rDNA was amplified based on the custom degenerate primer pair 1,106 (5′-TTWAGTCAGGCAACGAGC-3′) and barcode-1378 (5′-TGTGCAAGGAGCAGGGAC-3′) (Watanabe, Kimura & Asakawa, 2006) to construct a library. The PCR (25 µL) was performed with a 10  ×   buffer (2.5 µL), dNTP (2.5 mM of each), primers (3.0 pmol of each), Taq DNA polymerase (0.5 U rTaq, TaKaRa, Dalian, China) and DNA template (∼50 ng). The PCR cycling steps were as follows: hot start at 95 °C for 3 min, followed by 28 cycles of 94 °C for 30 s, 54 °C for 30 s, 72 °C for 30 s, and ending with a 72 °C extension for 10 min. To minimize PCR bias, each sample was amplified in triplicate, and the three samples were subsequently mixed together for sequencing.

Sequencing of the archaeal 16S r DNA was conducted using the MiSeq Illumina platform at Majorbio Biotechnology Company (Shanghai, China). Sequences were screened (<200 bp, no chimeras) from the resulting data with routines by Usearch (vsesion 7.0) for quality control. Sequences were aligned with the Silva database (http://www.arb-silva.de/) and subsequently analyzed using QIIME (version 1.7.0, Caporaso et al., 2010). The cutoff of the operational taxonomic units (OTUs) was at the 97% similarity level, and this resulted in 7153 OTUs that were clustered using Uclust (Edgar, 2010). Each OTU was annotated in a taxonomic way using the Ribosomal Database Project (RDP) classifier (Cole et al., 2007) based on a representative sequence and a training set that was extracted from the Silva108 database (Quast et al., 2012). All Illumina sequence data reported in this paper have been deposited in the NCBI Sequence Read Archive (SRA) database (accession no. SRP145544).

### Statistical analysis

The differences between values of the mean metal concentration, methane production, and gene abundance in the different treatments of this study were statistically analyzed by one-way analysis of variance (one-way ANOVA) and considered significant at the level of *p* < 0.05, and the Duncan test was used for post hoc analysis; all analyses were conducted using SPSS for Windows.

## Results

### Fractionation analysis of nickel and cobalt in paddy soil

**Figure 1:**
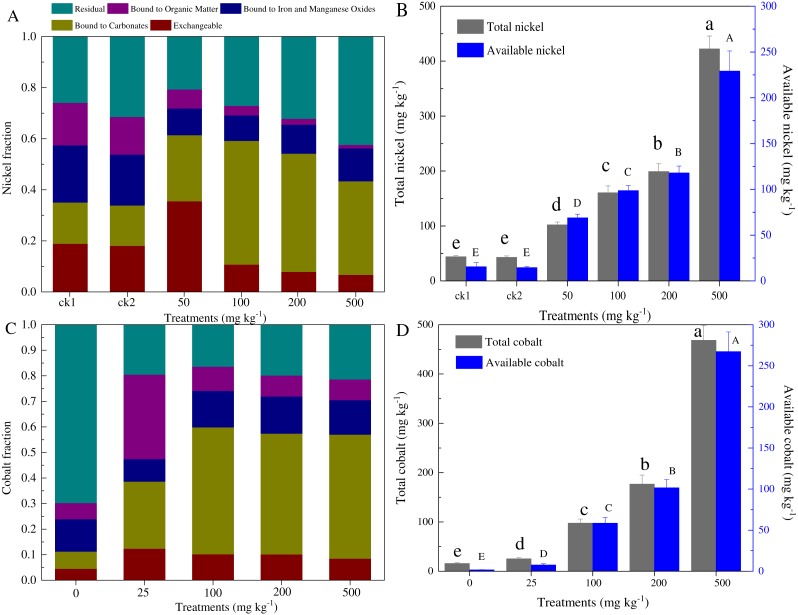
Partitioning of Ni and Co among the five fractions distinguished by our analysis (A, C), and total and available amounts of Ni and Co (B, D) in the soils with different levels of heavy metal amendments. ck1 and ck2 indicate the control without Ni addition with and without acetate addition, respectively. The lower and capital letters indicate significant differences between treatments at *p* < 0.05 (Duncan’s ANOVA) for the total and available Ni and Co, respectively.

According to the Tessier method, five forms of Ni and Co in the paddy soil were measured (Fig. 1). The metal-exchangeable and carbonate-bound Ni accounted for 33.9–35.1% and 43.4–61.4% of the total Ni for control and contaminated soils, respectively. The total Ni generally equaled to that of the total Ni added (i.e., 43.0–422.4 mg kg^−1^ dry soil), while the available Ni content was distributed at 24.5–229.2 mg kg^−1^ dry soil (Fig. 1B). Similarly, the total Co concentration ranged from 15.8 to 468.4 mg kg^−1^ dry soil, and the available Co ranged from 1.78 to 267.3 mg kg^−1^ dry soil (Fig. 1D). The amounts of available cobalt, including the exchangeable and carbonate-bound cobalt, increased greatly, even with the low concentration cobalt additions (58.7–229.1 mg kg^−1^ dry soil).

**Figure 2:**
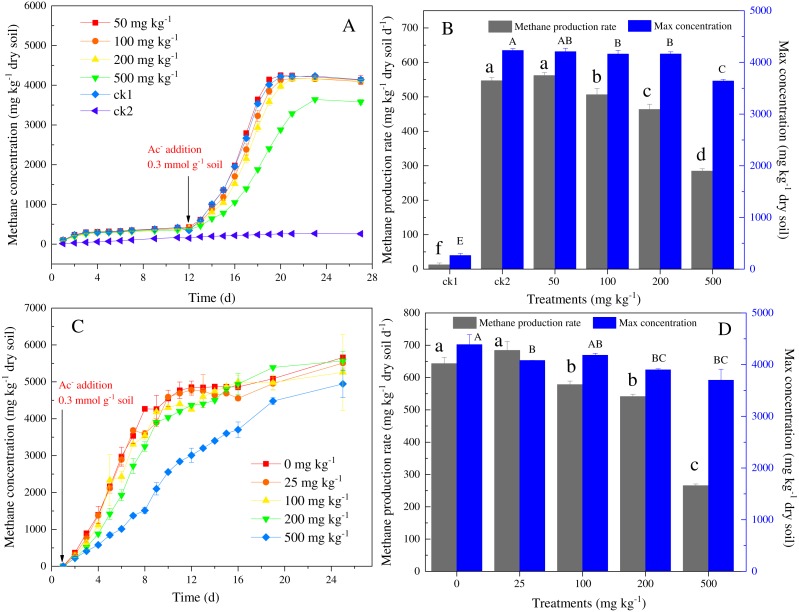
Temporal variations of methane concentration in headspace (A, C), and maximal CH4 production rates and concentration (B, D) in the incubation experiments for soils with different amounts of Ni (A, B) and Co (C, D) added. ck1 and ck2 indicate the control without Ni addition with and without acetate addition, respectively. The lower and capital letters indicate significant differences between treatments at *p* < 0.05 (Duncan’s ANOVA) for methane production and maximal concentration in Ni and Co, respectively.

### Effects of nickel and cobalt on kinetics of methane production in paddy soil

The effect of the addition of nickel on the methane dynamics in paddy is shown in Figs. 2A and 2B. Methane production occurred at very low levels (<500 mg CH_4_ kg^−1^dry soil) in the group without added sodium acetate and heavy metals (i.e., the control group, ck1) (Fig. 2A). The methane production rate (12 mg CH_4_ kg^−1^ dry soil d^−1^) and the maximum methane accumulation (266 mg CH_4_ kg ^−1^ dry soil) of the control group (ck1) were much lower than those of the other groups that had added sodium acetate (280–561 mg CH_4_ kg^−1^ dry soil d^−1^; 3,641–4,232 mg CH_4_ kg^−1^ dry soil) (Fig. 2B). Under anaerobic conditions, no distinct difference in methane production was observed between the treatments with different nickel concentrations during the initial preincubation stage (i.e., 0–12 days) (Fig. 2A). The methane production rate (561 mg CH_4_ kg^−1^ dry soil d^−1^) in the 50 mg kg^−1^ nickel addition was higher than that of ck2 (546 mg CH_4_ kg^−1^ dry soil d^−1^). However, the maximum methane accumulation was not significantly different. With the further increase in the nickel concentration, the methane production rate and maximum methane accumulation decreased to 284 mg CH_4_ kg^−1^ dry soil d^−1^. The methane production rates were significantly negatively correlated with the total nickel and the available nickel (*r*^2^ = 0.958, *p* < 0.05 and *r*^2^ = 0.899, *p* < 0.05, respectively), and the maximum methane accumulation was negatively correlated with the nickel content (*r*^2^ = 0.910, *p* < 0.01).

The effect of the addition of cobalt on the methane dynamics in paddy soil is shown in Figs. 2C and 2D. At the low concentration of 25 mg kg^−1^ of cobalt, the methane production rate (684 mg CH_4_ kg^−1^ dry soil d^−1^) and the lag-time of the methanogenic process showed no differences from the control group (643 mg CH_4_ kg^−1^ dry soil d^−1^). With the increase of the cobalt concentration, the generation of methane decreased by 45.4%–76.3%, and the lag-time also became longer. The methane production rates were significantly negatively correlated with the total cobalt and the available cobalt (*r*^2^ = 0.983, *p* < 0.05 and *r*^2^ = 0.981, *p* < 0.05, respectively).

### The *mcr*A gene abundance in contaminated paddy soil

**Figure 3:**
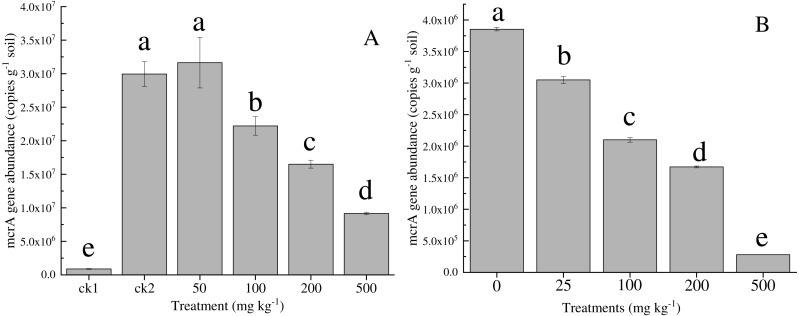
Abundance of mcrA gene in the soil with different amounts of Ni (A) and Co (B) added. Error bars indicate the standard deviation ( *n* = 3). ck1 and ck2 indicate the control without Ni addition with and without acetate addition, respectively. The different letters indicate significant differences between treatments at *p* < 0.05 (Duncan’s ANOVA).

The effect of nickel on the *mcr* A gene abundance of the methanogenic functional gene is shown in Fig. 3. The *mcr* A gene abundance (8.8  ×  10^4^ ± 0.8 × 10^4^ copies g^−1^ dry soil) of the control group (ck1) was two magnitudes lower than that in the other treatments that were supplemented with acetate substrates. The gene abundance (3.1  ×  10^7^ ± 0.5  ×  10^7^ copies g^−1^ dry soil) at 50 mg kg^−1^ was slightly larger than that of ck2 (2.2  ×  10^7^ ± 0.2  ×  10^7^ copies g^−1^ dry soil), but this difference was not significant. As the nickel addition increased to 500 mg kg^−1^, the abundance decreased significantly by one order of magnitude, to 9.2  ×  10^6^ ± 0.4  ×  10^6^ copies g^−1^ soil. Similarly, the abundance of the *mcr* A gene under the highest concentration of 500 mg kg^−1^ Co was one order of magnitude lower than that of the control.

**Figure 4:**
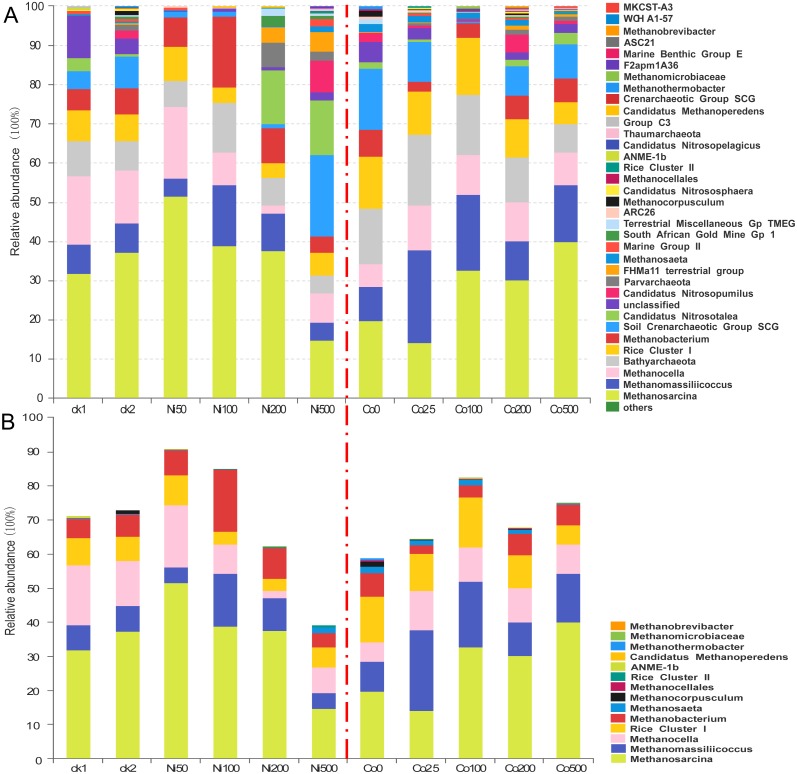
Composition of the archaeal communities (A) and methanogens communities (B) at genus level in the soils with different amounts of Ni and Co added. ck1 and ck2 indicate the control without Ni addition with and without acetate addition, respectively.

### Archaeal communities in paddy soil contaminated by nickel and cobalt

The archaeal compositions differed significantly at the genus level in the soils with different Ni and Co concentrations (Fig. 4). The most abundant *Methanosarcina* showed a relative abundance that varied from 14.5% at Ni500 to 51.3% at Ni50. In the Ni-amendment treatments, the relative abundance of *Methanosarcina* decreased with the increase of the nickel concentration above 50 mg kg^−1^. In the ck2 group (37.3%), which had added sodium acetate but no Ni additions, the *Methanosarcina* increased relative to the ck1 group (31.6%). Additionally, the relative abundance of *Methanosarcina* increased with the increase of the Co concentrations; at Co500, the increase was up to 39.8%. In treatments of all concentrations of Ni and Co, the other dominant species of archaea (1%–10%) included *Methanobacterium*, *Methanocella*, *Methanomassiliicoccus* and Rice Cluster I. The abundance of *Methanomassiliicoccus* was the highest under the treatment of Ni100 (15.0%), while that was the lowest under the treatment of Ni500 (4.26%). Low concentration of Co addition promoted the abundance of *Methanomassiliicoccus* (23.6% for Co25), which decreased with increasing Co concentration (9.52%–18.8%). The abundance of *Methanocella* ranged from 7.27% to 17.5% with Ni addition, except a low relative abundance (2.01%) observed at Ni200. With the addition of Co, the relative abundance of *Methanocella* was higher (8.52%–15.3%) compared with the control (Co0, 5.01%). When the Ni concentration was higher than 100 mg kg^−1^, the relative abundance of Rice Cluster I was lower (3.26%–5.76%) than that in the control and low concentration of Ni (7.02%–8.77%). With Co addition, the relative abundance of Rice Cluster I was much higher (9.27%–14.3%) except for the treatment Co500 (5.26%). The relative abundance of *Methanobacterium* ranged from 3.76% (Ni500) to 17.8% (Ni100) after Ni addition. Compared with the treatment Co0 (6.52%), the relative abundance of *Methanobacterium* decreased at low Co concentration (2.25%–3.01%), and increased with increasing Co concentration (5.76%–6.02%). *Methanocorpusculum* was observed in the control without the addition of metals (1.25% at ck2, ck), but not after the addition of nickel.

The relative abundance of the dominant genus *Bathyarchaeota* varied in the range 4.32%–12.31% for the Ni treatments and in the range 6.99%–17.8% for the Co treatments (Fig. 4A). Moreover, the lowest abundance was observed in the treatment with the highest metal additions (i.e., Ni500 and Co500), and the highest abundance was detected in Ni100 and Co25, respectively. The relative abundance of the soil Crenarchaeotic group (SCG) was 4.66% in ck1, 8.15% in ck2, and 20.80% in Ni500; however, SCG were not abundant in the Ni50, Ni100 and Ni200 treatments (<2%). Similarly, the SCG was abundant in all Co treatments (7.16%-15.6%) except Co100 (0.34%). In addition, we found that *Candidatus Nitrosopumilus* showed high relative abundances in the experimental groups with high amounts of metal, including Ni500 (8.32%) and Co200 (4.33%). *Parvarchaeota*, FHM a11 terrestrial group, South African Gold Mine Gp 1, *Candidatus Nitrososphaera*, *Candidatus Nitrosopelagicus*, Group C3, MKCSTA3, *Candidatus Methanoperedens*, and Rice Cluster II were observed at percentages less than 1% in the treatments with relatively high concentrations of added metals.

**Figure 5:**
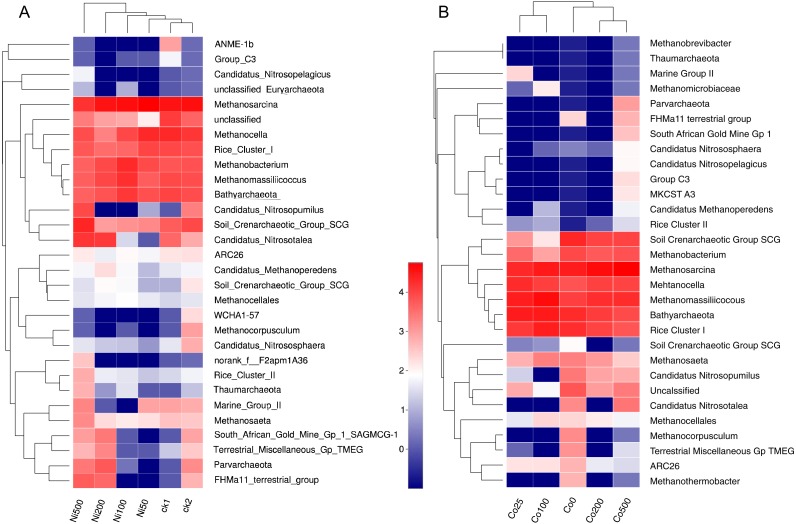
Heatmap analysis of the distribution of dominant phylotypes in soils with different amounts of Ni (A) and Co (B) added. Only the first thirty archaeal genera were considered. The top dendrogram is based on Euclidean distances according to the relative abundance of each genus. The relative percentages for the archaeal genera are depicted by the color intensity scale. ck1 and ck2 indicate the control without Ni addition with and without acetate addition, respectively.

The significant impacts of Ni and Co contamination on the archaeal community compositions were further demonstrated by the clustering of the dominant archaea genus corresponding to different Ni or Co levels in the heatmaps (Fig. 5). Similar patterns in shifts of the major archaeal genera were observed in the Ni and Co contamination. Generally, methanogens like *Mehtanosarcina, Methanocela, RC-I, Methanobacterium, Methanomassillicoccus* and *Batyarchaeota*, were dominant in the paddy soils and tended to have lower relative abundances at Ni500. *Nitrosopumilus* and *Nitrososphaera* were decreased with Ni addition. Soil Crenarchaeotic Group SCG, *Methanoperedens* and *Methanocellales* increased at low Ni concentration, and decreased at Ni500. Marine Group II and *Methanomicrobiaceae* increased at low Co concentration (Co25, Co100).

## Discussion

Iron (Fe), Ni and Co are the most demanded metals for microbial metabolic activity. As a trace element, Ni has been demonstrated to influence microbial growth, metabolism, community structure and diversity in methanogenic environments (Cai, Qiu & Chen, 2004; Paulo et al., 2017). Methanogenesis is considered one of the most metal-rich enzymatic pathways in biology. This study showed that Ni could play a key role in the activity and diversity of methanogens and other archaea. As one of the necessary trace elements for higher plants and some microorganisms, a lower concentration of Ni in soil can promote the growth and reproduction of microorganisms to a certain extent (Li et al., 2011). Our results showed that the addition of 50 mg kg^−1^ of Ni has neither stimulated nor inhibited the production of methane with the sodium acetate input, which might due to the background concentration of Ni in the investigated paddy soil (44.1 mg kg^−1^) and the concentration was higher than the average concentration of Ni in agricultural soils (30 mg kg^−1^) (Bennet, 1982). However, a toxic effect is observed above 50 mg kg^−1^ of Ni. It is known that heavy metal toxicity is one of the major causes for inhibition of anaerobic digestion (Chen, Cheng & Creamer, 2008; Chen et al., 2014). Nickel would cause 50% inhibition of mixed methanogens at 60 mg L^−1^ (Zayed & Winter, 2000). When the dissolved concentration of Ni was greater than 1 g m^−3^, methanogens were inhibited in an anaerobic digestion of sewage sludge (Ashley, Davies & Hurst, 1982). Similarly, Co is an important trace element for methanogenesis. However, the low bioavailability of Co has been reported to limit the growth of methanogens in both pure cultures and environmental studies. Therefore, it is concluded that methanogenic archaea require large amounts of Co (0.1–2 µmol, Glass & Orphan, 2012). Basiliko & Yavitt (2001) compared the CH_4_ production in peat from mineral-poor sites and mineral-rich sites by *in vitro* incubations with additions of trace metals (i.e., Fe, Ni, Co), and they found that the addition of metals enhanced this process in mineral poor sites and inhibited it in mineral rich sites. In the case of excessive Co concentrations, it would inhibit methanogens activity and growth. The optimum concentrations of Co for methane production in this study were 25 mg kg^−1^, both methane production and the abundance of functional gene decreasing when Co concentration was higher. Excessive heavy metals have been reported to inhibit soil microbial growth and reproduction and reduce microbial synthesis and enzyme secretion, eventually leading to reduced soil enzyme activity. Thus, low level of Ni and Co contamination could promote the growth and activity of methanogens in metal-limited paddy soil; on the other hand, high concentrations of metal pollution might affect the production and emission of greenhouse gases in paddy soil.

The toxicity of added Ni and Co is influenced by soil properties. Soil soluble metals are commonly considered to be the most active fractions, as they are available/toxic to soil organisms. These extractable fractions of metals are affected by soil chemical/physical properties including soil organic matter (SOM), soil pH, clay, reactive iron, aluminum, and manganese oxides (Fairbrother et al., 2007). Only a small fraction of the total metal present can be available (Hughes & Poole, 1991; Lemire, Harrison & Turner, 2013; Olaniran, Adhika & Balakrishna, 2013), and thus, it is critical to evaluate the metal availability in relation to the nutritional requirements of methanogens. In this study, the extractability and availability metals were assessed using sequential extraction, and we found that methane production was negatively correlated with the amounts of available Ni and Co, including the metal fractions exchangeable and bound to carbonates. Olaniran, Adhika & Balakrishna (2013) have reviewed the toxicity to microorganisms and the influence of metal speciation and bioavailability in soils, which is not addressed in most studies.

Different species of methanogens demonstrate unique responses to varying degrees of metal pollution, and these responses might be ascribed to different trophic types, abilities of metal acceptance and their affinities. Both acetoclastic and hydrogenotrophic methanogens identified in paddy fields have been shown to have high requirements of Ni and Co; these include *Methanosarcina*, *Methanospirillum*, *Methanocorpusculum*, *Methanobacterium* and *Methanococcus* species (Demirel & Scherer, 2011). Early studies showed that low concentrations of Ni, Co, and Mo in growth media could limit the growth of *Methanothermobacter thermautotrophicus* (Taylor & Pirt, 1977; Schönheit, Moll & Thauer, 1979). Furthermore, *Methanosarcinales* optimal growth was observed at low concentrations of Ni (0.1 µM) and Co (1 µM) (Scherer & Sahm, 1981; Lin et al., 1990). Our results found that the order *Methanosarcinales* had its higher relative abundances at 50 mg kg^−1^ Ni and 500 mg kg^−1^ Co.; these results are in agreement with the results from pure cultures, for example with optimal concentrations of 50µmol Ni and 0.1 µmol Co for *Methanosarcina barkeri* (Scherer & Sahm, 1981; Lin et al., 1990). This study found that *Methanobacterium, Methanomassillicoccus* and RC-I were stimulated at low Ni concentration, but were inhibited at high Ni concentration. In a pure culture study, *Methanobacterium* was reported to be quite resistant to Ni (Jarrell, Saulnier & Ley, 1987), regardless of the various concentrations of the added metals. In this study, we observed the abundance of *Methanobacterium* was increased 5 times at 100 mg kg^−1^ Ni compared with control, which indicated that *Methanobacterium* was relatively resistant to Ni. The relative abundance of non-euryarchaeotal methane-metabolizing lineages of *Bathyarchaeota* was comparatively stable across the various concentrations of Ni and Co; additionally, their relative abundance was higher in the Co treatments than in the Ni treatments. These observations lead us to formulate the hypothesis that *Bathyarchaeota* species might be more tolerant than *Euryarchaeal* species methanogens. Furthermore, *Bathyarchaeota* might play an important role in metabolizing methane in paddy soil polluted with heavy metals. In summary, the impact of CH_4_ production in the soil polluted with heavy metals was related to the responses of specific methanogenic archaea populations, and *Methanobacterium* and *Bathyarchaeota* were relatively resistant to Ni and Co in paddy soil.

Methanogens are abundant in paddy soil, and they have been recorded to range from 10^2^ to 10^7^ g^−1^ dry soil (Mer & Roger, 2001). With the addition of Ni and Co, *mrc*A gene abundance increased slightly at low concentrations of both Ni and Co amendments; however, *mrc*A gene abundance decreased significantly by one order of magnitude under the highest concentration of 500 mg kg^−1^ Ni/Co, which was in accordance with the methane production potential. These results indicated that high concentrations of Ni and Co affected the growth, proliferation and activity of methanogens.

There are various environmental factors, including nutrients, temperature, and physicochemical soil properties, that affect the emission of CH_4_ from paddy soil; this influence is ultimately due the effect of those parameters towards the microbial diversity and activity since those are correlated with methane production and consumption (Mer & Roger, 2001). It has been speculated that heavy metals impact both methane production and oxidation; however, the effects and mechanisms are inconsistent and complex for various metals, functional group taxa, soil textures and environmental conditions. Thus, in view of the large contribution of paddy soils to methane emissions and their increasing exposure to heavy metal pollution, the application of remediation practices in paddy soils should attempt to minimize both the toxic risk of heavy metals and their effects on greenhouse gas emissions. For example, as a sustainable remediation approach, biochar amendments would suppress the emission of CH_4_ and the bioavailability of heavy metals (Ibrahim et al., 2017). However, recent studies have explored novel microbial processes in the CH_4_ cycle, including anaerobic methane oxidation and aerobic methane production; the balance between these processes plays a key role in regulating methane emissions. Although heavy metals are generally believed to inhibit CH_4_production, there are still uncertainties regarding the contributions of paddy soils to the CH_4_budget when the paddy soils are polluted with various species and concentrations of heavy metals.

## Conclusion

Ni and Co pollution impacted the archaea communities in paddy soil; specifically, Ni and Co promoted the abundance and activity of methanogens at low concentrations, but they decreased both at high concentrations. The optimal concentrations of Ni and Co for methane production in paddy soil were 50 mg kg^−1^ and 25 mg kg^−1^, respectively. The dominant genus *Methanosarcinales* was sensitive to metal availability in paddy soil, and this genus reached its highest relative abundance in the Ni50 treatment; additionally, its relative abundance increased as the Co concentration increased to Co500. In view of the widespread contamination of paddy soils, our results could serve as a basis to guide future assessments of greenhouse gas fluxes from paddy soil polluted by Ni and Co.

##  Supplemental Information

10.7717/peerj.6274/supp-1 Data S1 Co raw data

10.7717/peerj.6274/supp-2 Data S2 Ni raw data

## Additional Information and Declarations

### Data Availability

The following information was supplied regarding data availability: Data can be found at the NCBI Sequence Read Archive: SRP145544.
